# Mitogen-Activated Protein Kinase and Exploratory Nuclear Receptor Crosstalk in Cancer Immunotherapy

**DOI:** 10.3390/ijms241914546

**Published:** 2023-09-26

**Authors:** Elke Burgermeister

**Affiliations:** Department of Medicine II, Medical Faculty Mannheim, Heidelberg University, Theodor-Kutzer-Ufer 1-3, D-68167 Mannheim, Germany; elke.burgermeister@medma.uni-heidelberg.de; Tel.: +49-621-383-2900

**Keywords:** MAPK, kinase, nuclear receptor, metabolism, xenobiotics, cancer, immunotherapy

## Abstract

The three major mitogen-activated protein kinase (MAPK) pathways (ERK1/2, p38, and JNK/SAPK) are upstream regulators of the nuclear receptor superfamily (NRSF). These ligand-activated transcription factors are divided into subclasses comprising receptors for endocrine hormones, metabolic compounds (e.g., vitamins, diet), xenobiotics, and mediators released from host immune reactions such as tissue injury and inflammation. These internal and external cues place the NRSF at the frontline as sensors and translators of information from the environment towards the genome. For most of the former “orphan” receptors, physiological and synthetic ligands have been identified, opening intriguing opportunities for combination therapies with existing cancer medications. Hitherto, only preclinical data are available, warranting further validation in clinical trials in patients. The current review summarized the existing literature covering the expression and function of NRSF subclasses in human solid tumors and hematopoietic malignancies and their modulatory effects on innate (e.g., macrophages, dendritic cells) and adaptive (i.e., T cell subsets) immune cells, encouraging mechanistic and pharmacological studies in combination with current clinically approved therapeutics against immune checkpoint molecules (e.g., PD1).

## 1. Introduction

**Figure 1:**
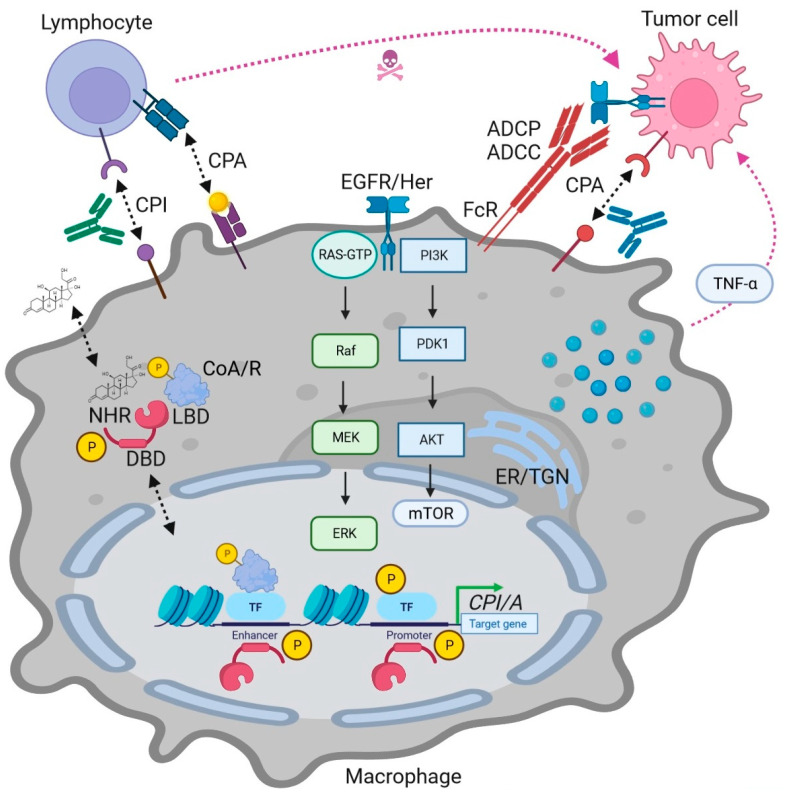
MAPK and NR interactions in cancer immunotherapy depicting modes of actions (MOAs) of NR ligands in immune cells. Genomic MOA: Ligands bind to intracellular NRs together with coactivators (CoA) or corepressors (CoR), followed by binding to DNA-response element in promoters or enhancers of target genes to activate or repress transcription in cooperation with other transcription factors (TFs, e.g., NFκB), respectively. MAPKs (and other kinases) phosphorylate (“P”) NRs, coregulators, and transcription factors to fine-tune transcriptional events. Non-genomic MOAs (not shown) as described in the companion article on endocrine (“hormone”) NRs have yet to be identified and validated for exploratory and “exotic” metabolic NRs; nonetheless, receptor tyrosine kinase (RTK)-MAPK pathway-dependent serine/threonine-directed phosphorylation has been reported. In immune cells, exemplified here by lymphocytes (T cells) and antigen-presenting cells (macrophages), NR ligands and their receptors alter expression of inhibitory (CPI) and activatory (CPA) immune checkpoint genes (e.g., PD1, PDL1, CTLA4) and soluble factors (e.g., chemo/cytokines). Pharmacological and genetic intervention with RTK-MAPK signaling by blocking Abs (e.g., against EGFR/Her) or NR agonists/antagonists (Table 1) can enhance recognition and elimination of tumor cells by immune cells. Abbreviations: Ab = antibody (depicted as Y-shaped structure); ADCC = antibody-dependent cellular cytotoxicity; ADCP = antibody-dependent cellular phagocytosis; ER/TGN = endoplasmic reticulum and trans-Golgi network; FcR = Fc receptor (for Abs bound to tumor cell antigens); P = phosphorylation (e.g., by MAPKs).

**Figure 2:**
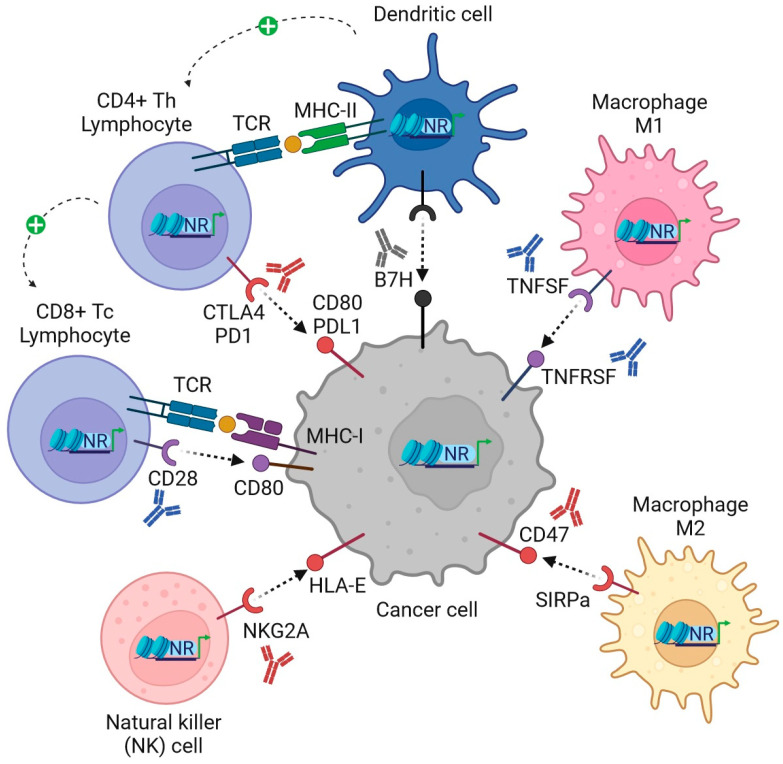
Model for novel combinations targeting the NRSF with clinical antibodies (Abs) in cancer patients. NRs and their ligands can inhibit expression of inhibitory (CPI) and activate expression of activatory (CPA) checkpoint genes in both tumor and immune cells, thereby enhancing immunogenicity, recognition, and elimination of tumor cells by immune cells. Legend (clockwise): In CD4+ T-helper lymphocytes (Th), pharmacological or genetic modulation of CPI expression (e.g., PD1, PDL1, CTLA4) and their respective counterpart molecules on cancer cells allows synergies with current clinically-in-use CPI blocking Abs (e.g., anti-CTLA4 ipilimumab, anti-PD1 nivolumab/pembrolizumab, anti-PDL1 atezolizumab/avelumab, e.a.) to boost the T cell receptor (TCR)-MHC class II “helper” synapse. In dendritic cells (DCs), agonistic or antagonistic Abs against members of the B7H family hold the promise to augment antigen presentation towards Th cells. In macrophage subsets, ranging from anti-tumor/pro-inflammatory (M1) towards immunosuppressive/anti-inflammatory (M2), phenotypes can be reshaped by agonistic Abs (e.g., against members of the TNF receptor superfamily TNFRSF, e.a.) or blocking Abs (e.g., magrolimab against the “do not eat me” signal CD47). In natural killer (NK) cells, blockage of CPIs (e.g., NKG2A by monalizumab) reduces immunosuppression by stress/tumor-driven MHC class I antigens (e.g., HLA-E; MICA/B, e.a.). In CD8+ T-cytotoxic lymphocytes (Tc) receiving Th “help” (marked by a green + ”plus” sign), targeting of CPAs (e.g., CD28, ICOS, CD40, e.a.) by agonistic Abs (e.g., sotigalimab/selicrelumab against CD40) has been designed to enhance co-stimulation within the TCR-MHC class I “killer” synapse. Color legend: blue Ab = agonistic/stimulating/activating Ab; red Ab = antagonistic/blocking/neutralizing Ab (Abs are depicted as Y-shaped structures).

Mitogen-activated protein kinases (MAPKs), comprising ERK1/2, p38, and JNK/SAPK, among others, phosphorylate most, if not all, members of the nuclear receptor superfamily (NRSF) [1]. A prime example was given by estrogen receptor(s) in breast cancer patients and their molecular, cellular, and in vivo functions [2] both in rodent models and humans, as detailed in the current issue’s companion article (https://doi.org/10.3390/ijms241713661; accessed on 4 September 2023). The clinical and preclinical utility of targeting endocrine and metabolic receptors has been confirmed over many decades, and recently also in a definite number of clinical trials in combination therapies with immune checkpoint antibodies (Abs) (e.g., PD1, PDL1, and CTLA4), both for solid tumors and hematopoietic malignancies [3]. Moreover, for many former “orphan” receptors of the NRSF, multiple natural and synthetic ligands (agonists, antagonists, and inverse agonists) have been identified (Table 1), adding novel opportunities for drug targeting [4]. Here, predominantly metabolic and xenobiotic receptors play a role as sensors and modifiers of the innate and adaptive host immune response against infection by pathogens, chemical insults, and tissue injury [5]. In the current article, I describe the expression and function of these “exploratory” NRs (Figure 1) and their potential to be eligible for future combination with immune checkpoint therapies in patients with cancer (Figure 2).

## 2. Exploratory Metabolic Receptors

### 2.1. Peroxisome Proliferator-Activated Receptor Beta/Delta (PPARB/D)

In contrast to PPARα/γ, whose applications and challenges have been extensively discussed in the companion article, selective targeting of PPARβ/δ for the metabolic syndrome has not given sufficient clinical benefit and was discontinued. Like PPARα, this isoform regulates target gene profiles, promoting energy expenditure and fatty acid oxidation, but has been implicated in promoting carcinogenesis in preclinical studies as well. As such, the phase 2 study with the selective PPARδ agonist seladelpar (MBX-8025) was terminated due to toxicity in patients with ursodeoxycholic acid-resistant primary biliary cholangitis [6].

Therefore, preclinical data argue for caution. The synthetic PPARδ agonist (GW501516) [7] evoked rapid progression of K-RAS mutant/PPARδ+ pancreatic intraepithelial neoplasia precursor lesions to ductal adenocarcinoma in mice. Herein, PPARδ agonists and/or high-fat diets induced K-RAS mutant pancreatic epithelial cells to synthesize the chemokine CCL2, leading to the recruitment and infiltration of macrophages and myeloid-derived suppressor cells (MDSCs). This CCL2/CCR2 axis drives an immunosuppressive tissue microenvironment, which may be targeted by PPARδ antagonists to prevent lethal pancreatic cancer.

A landmark study confirmed that PPARδ undermines anti-tumor immunity and promotes immune evasion in murine models of pancreatic cancer [8]. Therein, the mitochondrial enzyme glutamic-oxaloacetic transaminase-2 (GOT2) bound to fatty acid ligands and stimulated PPARδ’s transcriptional activity, leading to the spatial exclusion of CD4+CD8+ T cells from the tumor tissue.

As valid for PPARα, metabolic reprogramming of natural killer (NK) cells by administration of fatty acids or PPARδ agonists restricted anti-tumor responses in obese melanoma-bearing mice [9].

Conclusively, PPARδ seems to work as a tumor promoter and may call for the development of selective antagonists to fight cancer.

Direct genomic actions of PPARδ are exemplified in [10] human monocyte-derived macrophages, where PPARδ agonists repressed canonical target genes involved in metabolism and inflammation (NFκB, STAT1), whereby an anti-inflammatory IL4+ M2 phenotype was induced. Concomitantly, PPARδ agonists also augmented macrophage survival under hypoxia as well as CD8+ T cell activity, followed by repression of immune checkpoint genes PDL1, CD32B inhibitory Fcγ receptor IIB, indoleamine-2,3-dioxygenase-1 (IDO1), and its metabolite kynurenine, alluding to the potential of targeting PPARδ to rewire immune responses.

Accordingly, PPARδ agonist (GW501516) abolished the IL6-driven acute phase reaction in human hepatoma cells and rat primary hepatocytes by inhibition of STAT3 [11], preventing systemic inflammation via reduction in mRNA expression (α1-anti-chymotrypsin, α2-acid glycoprotein, β-fibrinogen, α2-macroglobulin) and secretion of C-reactive protein, whereas genetic depletion of PPARδ reversed these effects. Mechanistically, ectopic PPARδ or PPARδ agonists abrogated IL6-induced binding of STAT3 to the promoter of the α1-anti-chymotrypsin gene, underscoring the anti-inflammatory role of this NR.

MAPK-specific genomic interactions have been examined. In the skin, PPARδ drives H-RAS-mediated cellular senescence and inhibits tumorigenesis in mice [12]. PPARδ increased phosphorylation of ERK1/2 through an H-RAS-driven feedback mechanism, leading to transcriptional up-regulation of the RAS-guanyl-releasing-protein-1 (RASGRP1) gene. AKT inhibition by PPARδ was possible by repression of genes encoding for upstream AKT-activating enzymes (ILK, PDK1), followed by induction of senescence genes (P53/P27). PPARδ is also correlated with senescence phenotypes in human benign neurofibromas and colon adenomas. Since “senescence-associated secretory phenotype” (SASP) is of major importance for the biology of tumor-associated macrophages to generate an immunosuppressive tissue niche, targeting this NR may be of therapeutic utility.

Non-genomic crosstalk was described as follows [13]. PPARδ-deficient murine bone marrow and peritoneal mast cells had reduced amounts of high-affinity IgE receptors (FcεRI), less dense granules containing enzymes and proteases, and an altered cytokine profile. Mechanistically, loss of PPARδ resulted in modulation of PLCγ1 and ERK1/2 activities, affecting downstream degranulation and secretory phenotypes of mast cells.

In high-fat diet-fed mice, PPARβ/δ agonists [14] stimulated a rapid autocrine forward loop of stress-response cytokine (GDF15) and AMPK activation to ameliorate metabolic performance, endoplasmic reticulum stress, and inflammation, suggesting concomitant activation of p38 and JNK MAPKs.

Similarly, in chronic B-lymphocytic leukemia [15], PPARδ triggered JAK-mediated STAT phosphorylation, followed by enhanced production of IL10/IFNβ, cholesterol, and plasma membrane components, an effect that was reversed by PPARδ antagonists. Thereby, up-regulation of costimulatory molecules was prevented, indicating failure of the anti-cancer host immune response.

To date, no bona fide non-genomic receptors have been identified for PPARδ or its ligands, except for those discovered for free fatty acids (e.g., GPR40/120, e.a.) and the pan-PPAR post-translational modifications [16]. Notably, in human colorectal cancer cell lines, the EGFR-induced tyrosine phosphorylation of PPARδ led to the recruitment of HSP90, and its stabilization conferred chemoresistance to the EGFR inhibitor gefitinib [17].

Finally, transcriptional and non-transcriptional functions of PPARβ/δ can be combined [18]. For example, in human non-small-cell lung cancer cell lines, induction of vascular endothelial growth factor (VEGF) was caused by PPARδ binding to the *VEGF* promoter, among others (*COX2, cPLA2, PGES*), together with activation of PI3K-AKT signaling. Mechanistically, PPARδ interacted with the p85α regulatory subunit of PI3K to increase cell proliferation and survival, inflammation, and angiogenesis, highlighting the idea of PPARδ antagonism to abolish both modes of action.

### 2.2. Liver X Receptor (LXR)

LXRs (α/β) are regulators of lipid (cholesterol) and glucose homeostasis. Natural derivatives of cholesterol (oxysterols) and synthetic ligands were proposed as medications against hyperlipidemia and atherosclerosis. So far, no LXR-targeting drug has been approved for clinical use, in part due to unwanted side effects on lipogenesis.

A preclinical landmark report demonstrated that LXRs exert anti-inflammatory actions to govern host immunity, especially in macrophages [19]. Ligand-treated murine macrophages stimulated with endotoxins or live bacteria (*Escherichia coli*) up-regulated lipogenic genes but down-regulated pro-inflammatory ones encoding for nitric oxide synthase (NOS), cyclooxygenase-2 (COX2), and IL6. In mouse models of experimental contact dermatitis and atherosclerosis, LXR agonists mitigated inflammation, suggesting this NR as a target at the crossroad of metabolism and inflammation.

Whether LXR modulation is relevant for cancer immunotherapy remains to be proven. Nonetheless, inverse agonism of LXRs allows tumor elimination by enforcing the activity of CD8+ T cells in mouse and human cell models of triple-negative breast cancer [20]. LXRα/β expression was elevated in immunosuppressive tumor-associated myeloid cells. In contrast, pharmacological inhibition of LXR inhibited tumor growth via activation of cytotoxic CD8+ T cells and mitochondrial metabolism, emphasizing the potential of LXRs in novel strategies to reprogram the tumor tissue microenvironment.

Consistent with the benefit of LXR antagonism, LXR activation reduced the expres-sion of CC-chemokine-receptor-7 (CCR7) on dendritic cells (DCs) and impaired the anti-tumor response in mice [21]. Notably, tumors secrete natural sterole-based LXR agonists and thereby inhibit chemotaxis (“homing”) of DCs to tumor-draining lymph nodes. Mice with xenografts expressing sulfotransferase-2B1b (SULT2B1b), an enzyme that inactivates LXR ligands, restored DC infiltration and intratumoral inflammation and regained tumor surveillance. Genetic depletion of LXRα generated a similar phenotype. Since human cancer tissues are also present with CD83+CCR7- DCs, LXR inhibition may improve the tumor milieu in patients as well.

Hitherto, direct genomic actions of LXRs on immune checkpoint genes have not been reported. However, in murine and human macrophages, LXR agonists [22] together with stimulants of toll-like receptors (TLRs) induced *IL1B/Il1b* mRNA via activation of hypoxia-induced-factor-1-α (HIF1α) and related pathways (e.g., glycolysis). Homogenates from atherosclerotic carotid plaques achieved a similar effect, underscoring that LXR inhibition may also attenuate inflammation in patients.

A study on mouse melanoma examined the mechanism by which adoptive cell transfer with IL9+CD8+ cytotoxic T cells (Tc9) elicits a stronger anti-tumor response than with classical ones (Tc1) [23]. Overall, cholesterol and its derivatives inhibited IL9 expression by activating LXR resulting in its sumoylation and reduced binding of NFκB (RELA/p65) to the *Il9* gene promoter. Vice versa, Tc9 cells had lower cholesterol levels than Tc1 cells and, hence, elevated IL9, which fosters Tc9 cell persistence and anti-tumor efficacy. This link of a key nutrient, cholesterol, to cancer immunosurveillance may be exploited in the future in combination with checkpoint therapies.

Non-genomic crosstalk with components of the MAPK pathway was described for LXR [24]. In genetic, xenograft, and diet-induced mouse models of hepatocellular carcinoma, LXR agonists and RAF inhibitors counteracted therapy resistance. LXRα-driven lipogenesis and RAF1 inhibition acted in a synthetic lethal mode, where tumor cells succumbed to excess deposition of unsaturated fatty acids. Mechanistically, RAF1 is bound to and activates stearoyl-CoA-desaturase-1 (SCD1). Allosteric RAF1 inhibitors (e.g., BI882370) disrupted this complex and thereby promoted lipotoxicity, proposing metabolic targeting of liver cancer for patients as well.

Transgenic mice [25] with constitutive active NeuT/ErbB2 and fat-inducible caspase-8 suffer from loss of mammary fat and progressive deposition of the extracellular matrix, resulting in fibrosis and breast cancer. In this inflamed and immunotolerant tumor milieu, pan-LXR agonist DMHCA (N,N-dimethyl-3-β-hydroxy-cholenamide) attenuated tumor growth, desmoplasia, and fibrosis. LXR activation diminished infiltration of MDSCs and increased CD4+CD8+ effector T cells (Teffs) by lowering cancer-related gene expression (*Spp1*,* S100a9*,* Anxa1*,* Mfge8*,* Cd14*), suggesting LXR agonism to boost immune control in fibrotic tumors.

In mouse bone marrow-derived macrophages, LXR represents a downstream target of PI3K-AKT-mTOR signaling [26]. Specifically, the synthesis of natural LXR ligands (e.g., 25-hydroxycholesterol) depended on the lysosomal adaptor protein Lamtor1, which forms an amino acid sensor complex with lysosomal vacuolar-type H+ATPase and mTORC1, promoting M2 polarization of macrophages. Vice versa, *Lamtor1* gene depletion, starvation, or ATPase/mTOR inhibition favored M1 polarization.

Taken together, the rational design of combination therapies of kinase inhibitors and LXR modulators may exploit lethal anti-tumor circuits at the interface of metabolism and immunity.

Recently, non-genomic effects of LXR ligands have been detected, with the contributing receptors yet to be identified. As such, LXR agonists (T0901317, GW3965) exerted multiple and rapid NR-independent effects on insulin secretion in pancreatic β-cells of mice [27].

Likewise, serine-directed phosphorylation of LXR impacts both the function and localization of the NR [28]. In mice, LXRα phosphorylation ameliorates atherosclerosis by altering macrophage proliferation, polarization, and effector functions (e.g., phagocytosis, efferocytosis) and the oncogenic transcriptome (FOXM1, e.a.) [29], indicative of LXR antagonism as a potential target to improve host metabolism and immune performance.

### 2.3. Nuclear Receptor Subfamily 1 Group D Member 1/2 (REVERBA/B)

REVERBs (α/β) function as bona fide transcriptional repressors via recruitment of histone deacetylases (HDACs) and NR corepressors within the mammalian circadian rhythm clock. In addition to synchronizing and maintaining endocrine and metabolic timing (e.g., as heme/redox sensors) and homeostasis, they also impact tissue regeneration (e.g., by DNA damage repair) and immunity. In humans, disrupted rhythms (e.g., by jet lag or night shift work) are associated with metabolic, cardiovascular, and eventually malignant diseases.

Hitherto, stenabolic (SR9009) is available off-label for muscle mass doping. However, whether REVERB ligands are suitable for prevention or clinical intervention has yet to be proven. Previous preclinical studies in mice led to the concept that circadian behavior and metabolism can be directly targeted by genetic or pharmacological modulation of REVERBs [30,31].

Experimental evidence regarding cancer immunotherapies is still emerging. In mouse models of chronic pancreatitis [32], genetic perturbation of the circadian master clock circuits (BMAL1/CLOCK, RORA/REVERBA) resulted in TGFβ-IL11/IL11RA-dependent progressive pancreatic fibrosis and exocrine dysfunction due to the fibrogenic properties of pancreatic stellate cells and secretory activity of acinar cells. Importantly, pharmacological restoration of the circadian clock by the combination of melatonin with the RORα agonist SR1078 attenuated the pathological alterations in the mouse pancreas, suggesting a protective role of clock proteins in organ function also for humans.

A landmark study revealed that [33] REVERBα/β agonists (SR9009, SR9011) are lethal for human and murine cancer cells and oncogene-induced senescent cells, including melanocytic naevi. Mechanistically, induction of autophagy, lipogenesis, and apoptosis converged to exert anti-tumor effects in the presence of hypoxia, oncogenic driver genes (H-RAS, B-RAF, and PIK3CA), and p53 deficiency. In mouse glioblastoma models, tumor growth was reduced and animal survival prolonged, confirming that pharmacological modulation of the circadian machinery has the potential to impose clinical benefit.

Direct genomic actions on immune-relevant genes have been described. As such, REVERBα knockout mice [34] suffer from severe chemically induced colitis through activation of the NLRP3 inflammasome, an effect that was reversed by REVERBα agonist (SR9009) in wildtype (wt) littermates. REVERBα bound to the promoters of the *Nlrp3* and *P65/RelA* genes to repress transcription, implicating that REVERBα may be suitable in the prevention or treatment of inflammatory bowel disease.

Consistent with this anti-inflammatory action, REVERBs inhibit distal enhancers selectively bound by macrophage-lineage-determining factors (PU.1, C/EBP, AP1) [35]. Mechanistically, REVERBs inhibited the transcription of enhancer-driven eRNAs and thereby repressed locally adjacent mRNAs involved in macrophage differentiation and effector functions (MMP9, CXCR3), underscoring the role of these receptors in the regulation of host immunity.

These data were confirmed upon macrophage-specific REVERBα and BMAL1 depletion in vivo and in vitro [36], where the temporal response to endotoxin-induced production of inflammatory cytokines was completely abolished in the knockout animals. REVERBα agonism (GSK4112), or genetic knockdown, reciprocally altered gene expression and secretion of IL6 in human macrophages, corroborating the immunosuppressive function of REVERBs.

REVERBα was also found to antagonize RORγt, an NR and major driver of pro-inflammatory Th17 cells involved in autoimmune diseases [37]. In Th17 cells, REVERBα bound ROR-responsive DNA elements and repressed transcription of RORγt-dependent genes (e.g., IL17A/F). Accordingly, the REVERBα agonist or its overexpression ameliorated the development of experimental autoimmune encephalomyelitis (EAE) in mice, proposing it as a potential future medication against immune-related adverse events (IRAE) in checkpoint-Ab-treated patients with cancer.

REVERBα activated mTORC1 signaling via transcriptional repression of the gene encoding for the mTORC1 inhibitor *Tsc1* [38], resulting in phosphorylation of BMAL1 and altered circadian rhythms in the mouse liver. Agonist-activated REVERBs (GSK4112) inhibited bone differentiation through p38 MAPK signaling in osteoclastic macrophages and osteoblasts [39]. Specifically, REVERBs interacted with tumor-relevant transcription factors (c-Fos, Runx, NFAT) at gene promoters (RANKL, alkaline phosphatase, e.a.) in vitro and in mice.

REVERBα agonist or its overexpression also prevented endotoxin-induced chemokine synthesis (e.g., *Ccl2*) in the murine macrophage cell line RAW264 [40]. REVERBα directly recognized a DNA-binding element in the *Ccl2* promoter and non-genomically blunted CCL2-triggered ERK1/2 and p38 activation, followed by decreased cell adhesion and migration. Vice versa, macrophages from knockout mice had elevated CCL2 levels and enhanced tissue infiltration rates, and animals with inflammatory conditions (aging, obesity) displayed down-regulation of *Reverba (Nr1d1)* expression.

Evidence for non-genomic NR-independent actions was collected for REVERB agonists [41] in double-knockout mice for the two *Reverba/b (Nr1d1/2)* genes. Therein, SR9009 reduced proliferation and viability, mitochondrial metabolism, and the transcriptomes of liver and embryonic stem cells. Likewise, SR9009 inhibited the growth of cell lines derived from the most lethal human prostate cancer subtype independently of REVERBs by blocking the LXRα/FOXM1 pathway [42], affecting colony formation, cell cycle, migration, and apoptosis. SR9009 also restrained tumor growth in human prostate cancer cell xenograft models, suggesting other NRs (e.g., LXRs) as potential targets of REVERB ligands.

A preclinical study proved that systemic restoration of circadian rhythms by administration of a cyclin-dependent kinase inhibitor (seliciclib) attenuated tumor growth in mice with osteosarcoma [43]. Mechanistically, CDK1/2/7/9, ERK1/2, and CK1 were identified as targets synchronizing the circadian with cell cycle rhythms. Notably, this drug revived rhythmic expression patterns in arrhythmic tumors, with positive effects on clock genes vs. suppressive ones on genes driving cell division (*c-Myc, Wee1*).

Recently, serine-directed phosphorylation of the REVERB’s N-terminal domain has been discovered to govern its intracellular localization, alluding to a general theme for most NRs [44]. Since REVERBs also locate to the cytoplasm or membranes dependent on their phosphorylation state [44], non-genomic functions may also be transduced by non-NR effectors, and the role of their physiological ligands, such as Fe^3+^/Fe^2+^ iron-bound heme and proto-porphyrines functioning as intracellular sensors for diatomic gases (CO/NO), has yet to be unveiled [45,46].

### 2.4. Retinoic Acid Receptor-Related Orphan Receptors (RORs)

RORs (*α/β/γ*) comprise a subset of three NRs, with RORA being one of the major components in the circadian clock, while RORB is a developmental regulator and RORC, respectively, its variant RORγt is a major driver of Th17 inflammatory responses [47]. The transcriptional activator RORα binds to the same DNA element (i.e., RORE) as REVERBα, antagonizing its function as a repressor [48]. Natural and synthetic ligands overlapping with RAR ligands have been identified and may be useful in regulating host immunity responses.

An outstanding clinical case report claimed that inherited PD1 deficiency underlies susceptibility to tuberculosis and lethal autoimmunity in children [49], clinically phenocopied by IRAE upon checkpoint inhibitor therapy with PD1 Abs. In this patient, PD1 leukocytes failed to release IFNγ upon infection with mycobacteria, were devoid of Vδ2+γδ T cells, mucosal-associated invariant T (MAIT), and CD56bright NK cells, and exhibited dysfunction of several T cell subsets. As such, the massive release of STAT3-activating cytokines (IL6/IL23) triggered the expansion of RORγt+CD4-CD8-αβ T cells, hepatosplenomegaly, and lymphoproliferative autoimmunity. However, if pharmacological modulation of the RORγt axis has benefits for cancer patients remains to be explored.

In view of cancer immunotherapy, preclinical studies in mice revealed that [50] hypoxia stimulated the secretion of glioma-derived exosomes, which are then taken up by MDSCs. Hypoxia-induced miR-10a/21 in exosomes, which negatively targeted RORα and PTEN expression, triggered the expansion and activation of MDSCs to secrete reactive oxygen species (ROS), nitric oxide (NO), arginase, IL10, and TGFβ, whereas miRNA knockout reversed this effect. Thus, activation of RORα may squelch the tumor immune environment into a less suppressive mode.

A landmark study demonstrated that commensal microbiota prevented food-borne allergies in mice [51]. Xenotransplants of human dysbiotic fecal microbiota with high levels of IgE > IgA failed, while *Clostridiales* with *Subdoligranulum variabile* or *Bacteroidales* protected recipient mice. Mechanistically, bacteria up-regulated RORγt in regulatory T cells (Tregs) via MyD88 signaling, promoting tolerance against external antigens and proposing a role for RORγt beyond Th17 biology.

In contrast, inhibition of RORγt limited intestinal inflammation in mice by reducing Th17 cells and preserving tissue-protective innate lymphoid cells (ILC3) [47]. In mice infected with *Citrobacter rodentium*, the RORγt inhibitor (GSK805) diminished the synthesis of pro-inflammatory cytokines in Th17 cells but not in ILC3, which are essential in maintaining epithelial homeostasis and pathogen defense, alluding to the potential for RORγt inhibition as a therapeutic target in inflammatory bowel diseases.

Restoration of RORγ expression reduced proliferation and glucose metabolism and increased apoptosis in cisplatin-treated bladder cancer cells in vitro and in vivo [52]. RORγ bound to and repressed the PDL1 gene promoter and blunted PDL1/ITGB6/FAK-dependent nuclear translocation of STAT3, proposing RORγ agonism as a novel anti-cancer approach in conjunction with checkpoint Abs.

Direct genomic actions on immune checkpoint genes are exemplified by synthetic vs. endogenous RORγt agonists [53]. The former foster proliferation and differentiation of Th17/Tc17 cells while inhibiting PD1 gene expression, followed by reduced Treg pools and enhanced synthesis of the pro-inflammatory cytokine IL17, predicting a good prognosis in cancer patients.

IL17 transcription [54] depends on a proximal promoter and a distal enhancer harboring DNA-binding motifs for RORγt and Runx1, where interaction between the latter two transcription factors abolished the inhibitory effect of FOXP3 on Th17 differentiation, indicative of a role of RORγt agonists in reshaping a tolerogenic tumor micromilieu.

MAPK-specific non-genomic crosstalk has also been shown for RORγ. IL1β-driven serine phosphorylation on RORγt by ERK1/2 limits hyperactivation of Th17 cells and promotes the synthesis of the anti-inflammatory cytokine IL10 [55]. Mice with a genetic knock-in of a phospho-null allele (RORγtS182A) suffer from more severe inflammation than wt littermates, both in models of colitis and EAE, proposing the IL1β-ERK1/2-RORγtS182 axis as a target to combat autoimmune disease or, vice versa, pronounce anti-tumor inflammatory phenotypes.

Likewise, RORα expression [56] is elevated in patients with inflammatory bowel disease resistant to treatment with TNFα neutralizing Abs. In mice, depletion of *Rora* in CD4+ T cells mitigated colitis by reducing infiltration and apoptosis of T cells. RORα evoked AKT-dependent mTORC1 activation both via transcriptional (e.g., on *Lamtor*) and post-transcriptional mechanisms; thus, inhibition of this signaling axis may be beneficial in patients as well.

RORα activated by agonist SR1078 also stimulated the expression of inflammatory cytokines in macrophages and adipocytes by provoking endoplasmic reticulum stress [57] in vitro and in mice. Mechanistically, SR1078 up-regulated mRNAs of stress response genes and enhanced phosphorylation of components within the “unfolded protein response” (PERK, IRE1α), again alluding to an overlay of non-genomic and genomic mechanisms, whose relevance for cancer immunotherapies remains open for investigation.

Similar to most NRs, serine-directed RORα phosphorylation by ERK1/2 [58] and other kinases [59,60] impacts its transcriptional and non-transcriptional activity. Again, non-NR receptors have not been identified yet. Instead, β-catenin signaling could be inhibited by WNT5a/PKC-dependent phosphorylation of RORα in murine colon cancer via a non-genomic mechanism [61].

Because of the promiscuous overlay of ROR, RXR, and RAR ligands, caution is warranted for the selective development of synthetic drugs, which are expected to prevent excessive inflammation and tissue damage but may preclude efficient anti-tumor host responses.

### 2.5. NR4A1 (NUR77), NR4A2 (NURR1) and NR4A3 (NOR1)

The NR4A subfamily of NRs belongs to the functional group of “immediate early” genes and is subjected to rapid transcriptional regulation and phosphorylation by a variety of stimuli, including cell stress, inflammation, and mitogenic cues, making them central sensors of a changing external and internal microenvironment in healthy and diseased host immunity. Putative ligands comprise poly-unsaturated fatty acids, prostaglandins, and synthetic compounds (such as cisplatin and cytosporone B). Thus, their clinical application demands further research.

A landmark study showed that NR4A receptors limit the function of chimeric-antigen-receptor-expressing T cells (CAR-T) in solid tumors [62]. To counteract the exhausted, dysfunctional state of host T cells upon chronic antigen exposure, human CAR-T cells directed against the B cell antigen CD19 were transplanted into mice bearing human CD19+ tumors. Therein, similar to tissue samples from patients with cancer or chronic virus infections, CD8+ CAR+ and endogenous CD8+ tumor-infiltrating T cells had high levels of inhibitory immune checkpoints (e.g., PD1, TIM3), driven by NFAT-mediated up-regulation of all three NR4A members. In contrast, *Nr4a* triple knockout mice achieved tumor control and survival. Mechanistically, NR4A DNA-binding sites at immunosuppressive gene loci were switched to immune activatory sites (e.g., AP1, NFκB) by chromatin remodeling to boost CD8+ effector T cell (Teff) function, proposing this receptor subclass as a potential target for prevention of T cell hyporesponsiveness.

Using a similar mouse model [63], NFAT was confirmed to induce expression of high-mobility group box (TOX/TOX2) and NR4A transcription factors in tumor-infiltrating, exhausted CD8+ PD1+ TIM3+ CAR-T cells. In contrast, *Tox* double-knockout CAR-T cells inhibited tumor growth and improved animal survival. Consistently, *Nr4a*/*Tox*-deficient CAR-T cells had fewer inhibitory checkpoints but more pro-inflammatory cytokines driven by DNA-binding motifs for NFκB and bZIP transcription factors, suggesting that NR4A antagonism may be a promising approach to rewire transcriptional networks at promoters of immune-relevant genes governing T cell functionality in cancer.

These data were confirmed by showing that transcription of all three *NR4A* genes was up-regulated in macrophages and fibroblasts by a variety of stimuli (e.g., endotoxin, cytokines, oxidized lipids) employing NFκB response elements in the murine and human promoter DNAs [64]. In contrast, genetic depletion of NR4A members [*Nr4a3* (*Nor1*); *Nr4a1* (*Nur77*)] promoted the development of lethal acute myeloid leukemia in mice [65], suggesting them as tumor suppressors. Therein, the rapid expansion of abnormal hematopoietic stem cells and myeloid progenitors was accompanied by the loss of stress-responsive transcription factors (AP1, JunB, c-Jun) and defective apoptosis (FasL, TRAIL). A similar loss of function was identified in patients, indicative of NR4A receptors as potential targets to prevent cancer.

As for other NRs, MAPK-specific non-genomic interactions comprise the post-translational phosphorylation of NR4A receptors, whereas non-NRs for rapid effects elicited by NR4A-specific agonists have not been described so far [66]. Consistently, mitogen-and-stress-activated-protein-kinases (MSKs) downstream of ERK1/2 or p38 are required for immediate early gene transcription of the NR4A gene family by CREB/ATF-driven promoters in murine embryonic fibroblasts [67], suggesting a reciprocal regulatory loop between the NRs and MAPKs.

Likewise, the direct genomic actions of all three members of the NR4A subfamily were stated.

For example, in human endothelial cells of atherosclerotic plaques, [68] inflammatory stimuli induced NFκB-dependent transactivation of the human *NOR1* promoter. In turn, NOR1 transactivated the promoters of cell adhesion molecules (VCAM1, ICAM1) by binding to NR4A-responsive DNA elements, followed by increased adhesion of monocytes. Consequently, *Nor1 ApoE* double knockout mice had fewer macrophage-loaded atherosclerotic lesions, proposing NR4A inhibition as a target to modulate macrophage infiltration.

Consistently, NURR1 positivity conferred a poor prognosis for patients with gastric cancer [69]. Therein, *Helicobacter pylori*-activated PI3K/AKT signaling triggered the Sp1 transcription factor to up-regulate the *NURR1* promoter. NURR1 in turn bound to and induced the *CDK4* promoter, proving an oncogenic loop for cancer cell proliferation in vitro and in mice. Virus-induced NURR1 also promoted cancer aggressiveness and radioresistance in human primary and cervical cancer cell lines [70]. NURR1 directly activated MEK1/2-ERK1/2, and PI3K-AKT-mTOR signaling by means of a so-far unknown non-genomic mechanism. In line with this data, apoptosis signal-regulating-kinase-1 (ASK1) and p38 elicited cytoplasmic translocation of phosphorylated NURR1 into the cytosol and triggered oxidative stress-induced necrotic cell death in vitro [71]. Moreover, NURR1 can be phosphorylated by ERK2 [72] in neuroblastoma cells, inferring potential relevance for cancer.

Specifically, phosphorylation of NUR77 by the MEK1/2-ERK1/2-RSK axis induces its nuclear export, mitochondrial translocation, and apoptosis in a murine T cell line [73]. In the latter cell line, NUR77 [74] is also phosphorylated by ERK5 (big MAP kinase 1, BMK1) downstream of the T cell receptor (TCR), leading to phospho-NUR77-dependent apoptosis, a pivotal event during the negative selection of autoreactive T cells hyperactivated by interaction with self-peptide–MHC complexes in the thymus. Vice versa, blockade of ERK5 signaling prevented TCR-NUR77-induced cell death, indicative of a role of MAPK-NR4A crosstalk in shaping the T cell repertoire and preventing autoimmunity. In contrast, AKT interacts with and phosphorylates NUR77 [75] within its DNA-binding domain (DBD), thereby decreasing its transcriptional activity and promoting cell survival in vitro.

A pivotal study on non-genomic actions elaborated [76] that NUR77 increased resistance to endotoxin-induced sepsis in mice by inhibiting NFκB-mediated expression of pro-inflammatory cytokines. Mechanistically, NUR77 formed a protein complex with p65/RELA to prevent its binding to NFκB-response elements, a life-protective interaction that was disrupted by endotoxin-induced p38 phosphorylation of NUR77. A synthetic compound (n-pentyl 2-[3,5-dihydroxy-2-(1-nonanoyl) phenyl]acetate) prevented p38-mediated phosphorylation by binding to the ligand-binding domain (LBD) of NUR77, rescuing protection against lethal hyperinflammation.

NUR77 was also identified as a transcriptional regulator [77] of pro-inflammatory metabolic reprogramming in murine RAW264.7 macrophages due to the skewing of isocitrate dehydrogenase (IDH) towards succinate dehydrogenase (SDH) activity. *Nur77* knockout macrophages produced higher levels of tricarboxylic cycle-derived metabolites (e.g., NO) and pro-inflammatory cytokines, exacerbating atherosclerosis in vivo, alluding again to the protective anti-inflammatory role of NUR77. NUR77 translocates from the nucleus to the mitochondria, where it associates with BCL2 proteins to induce apoptosis in human cancer cell lines. JNK activators, e.g., synthetic retinoid drugs (e.g., AHPC (E)-4-[3-(1-adamantyl)-4-hydroxyphenyl]-3-chlorocinnamic acid), phorbol ester, anisomycin, or MEKK1 triggered JNK-mediated NUR77 phosphorylation, its export to the cytoplasm, and apoptosis [78], whereas constitutively active PI3K/AKT signaling counteracted this effect.

Overall, post-translational modifications by MAPK pathways converge on the members of the NR4A family to specify their spatio-temporal transcriptional activities in determining host immunity, predisposing these NR as promising targets for future intervention trials in cancer patients.

### 2.6. Xenobiotic and “Exotic” Metabolic Receptors

A significant part of the NRSF comprises developmental regulators (e.g., GCNF, PNR, TLX, COUPTF, SF1, DAX1, TR2/4) which will not be considered therapeutic targets due to safety concerns. Nevertheless, several metabolic and xenobiotic NRs constitute potential new targets for cancer immunotherapy.

As such, liver-receptor-homolog-1 (LRH1) known to regulate glucose, bile acid, and lipid metabolism, is also required for T cell proliferation, maturation, and effector functions. LRH1-depleted CD4+ or cytotoxic CD8+ T cells fail to mount adaptive immune responses (e.g., during intestinal inflammation) or control viral infections [79]. Consistently, genetic or pharmacological inhibition of LRH1 restricted the endotoxin-induced synthesis of pro-inflammatory cytokines in murine macrophages [80]. Mechanistically, mitochondrial ATP release and metabolism via LRH1 targets (glucokinase, glutminase-2) were impaired. In a mouse model of hepatitis, LRH1 inhibition reduced TNFα synthesis and liver damage, suggesting LRH1 agonism as a booster for host immunity.

Mutations in the human hepatocyte-nuclear-factor-4α (*HNF4A)* gene are associated with a rare form of type 2 diabetes (MODY1) [81]. In mice, epithelial HNF4α is required for the development and homeostasis of intestinal epithelial cells [82]. HNF4α regulates the expression of genes (butyrophilin-like Btnl1/6, H2-T3, and Clec2e), allowing the expansion of natural TCRγδ+ or TCRαβ+ CD8αα+ intraepithelial immune cells. This HNF4A-BTNL regulatory axis was conserved also in humans, indicative of HNF4A agonism as a host immune activator. In hepatocellular carcinoma cells, HNF4A bound to and activated enhancer/promoter regions of the *HSD17B6* gene, a key enzyme involved in synthesizing dihydrotestosterone [83], resulting in the mitigation of tumor cell proliferation and invasion in vitro. This NR was also correlated with altered immune cell infiltration and checkpoint gene expression in patients.

A recent study discovered estrogen-related-receptor-α (ERRα) as a target for immunometabolic anti-tumor drugs in melanoma patients [84] using an in silico multiomics approach. Notably, ERRα was activated in tumors resistant to PD1 Ab, and its pharmacological inhibition killed tumors by suppressing energy metabolism and promoting M1 macrophage polarization via the release of pro-inflammatory cytokines and antigen presentation allowing infiltration of cytotoxic CD8+ T cells. ERRα also inhibited TLR-induced inflammation and metabolic reprogramming of macrophages [85]. ERRα-deficient mice were susceptible to endotoxin-induced septic shock, and *Esrra*(-/-) macrophages had higher glycolysis but impaired mitochondrial biogenesis and respiration. Mechanistically, ERRα bound to the promoter of *Tnfaip3*, a deubiquitinating enzyme in toll-like receptor signaling, and restricted NFκB activation by acetylation of p65/RELA.

ERRα is also a metabolic regulator of T cell activation and differentiation [86]. In Teffs, ERRα up-regulated glucose metabolism via the GLUT1 protein, whereas mitochondrial lipid oxidation was impaired, a process that fosters immunosuppressive Treg function. In vivo inhibition of ERRα reduced proliferation and generation of Teffs in EAE and immunization mouse models. In bone metastases from breast cancer, ERRα also improved anti-tumor immune responses [87] via the release of chemokines (CCL17, CCL20) and reduced synthesis of TGFβ3, allowing infiltration of cytotoxic CD8+ T cells and mitigating metastatic burden both in mice and humans, thus leaving the question open if agonism or antagonism of ERR is favorable for tumor patients.

NRs for xenobiotic compounds (e.g., CAR, PXR) may be repurposed for therapeutic use. For example, the constitutive androstane receptor (CAR) supports the response of the intestinal mucosa to injury [88]. CAR positivity was reduced in patients and mice with inflammatory bowel disease. CAR-deficient mice suffered from defective wound healing, which could be ameliorated by administration of 3,3′,5,5′-tetrachloro-1,4-bis(pyridyloxy) benzene (TCPOBOP), a selective CAR agonist. CAR activation also accelerated intestinal epithelial wound healing by cell migration in vitro, alluding to a role for agonists in strengthening host immunity.

Another report stated that CAR supports adaptation to bile acids in the murine small intestine [89]. In CD4+ Teffs, CAR protein up-regulated the expression of the xenobiotic transporter MDR1 (*Abcb1a*) to prevent bile acid toxicity and suppress intestinal inflammation. CAR also induced the synthesis of anti-inflammatory IL10 and detoxifying enzymes and transporters in Teffs and hepatocytes. Consistently, loss of CAR exacerbated ileitis, which was reversed by pharmacological CAR activators.

Finally, symbiotic bacterial metabolites stabilize the gastrointestinal mucosal barrier via the xenobiotic sensor pregnane X receptor (PXR) [90]. Mechanistically, microbial-derived indole 3-propionic acid as a natural PXR agonist down-regulated TNFα production in enterocytes but up-regulated mRNAs coding for cell junction proteins, thereby maintaining epithelial integrity. In contrast, PXR-deficient mice suffered from a “leaky” gut and activated TLR4 signaling, proposing PXR agonism as a protective treatment against inflammation and potentially inflammation-induced cancers. Accordingly, pharmacological PXR activation inhibited NFκB signaling, while PXR-deficient mice had increased expression of NFκB target genes and inflammation in the intestines [91]. Mechanistically, NFκB inhibited the transcriptional activity of PXR (e.g., on hepatic cytochrome P450 genes involved in xenobiotic detoxification) and, in turn, suggested a druggable PXR-NFκB axis to prevent infection and xenobiotic-induced inflammation, tissue damage, and immune cell dysfunction.

For those receptors, phosphorylation has been reported; however, the role of MAPK-dependent non-genomic crosstalk with immune checkpoint therapies still has to be explored. For example, phosphorylation by ERK1/2 of the hinge domain of LRH1 stimulates its transactivation function [92]. Likewise, p38 phosphorylates HNF4α to induce cholesterol-7α-hydroxylase, a key enzyme in bile acid synthesis [93]. PXR (rifampicin) and CAR (phenobarbital) agonists activate cytochrome P450 genes via phosphorylation of the DBD and LBD [94] by serine/threonine-directed kinases (ERK1/2, p38, GSK3β/AKT e.a. [95]). Of note, ERK1/2 up-regulated the expression, phosphorylation, and transcriptional activity of ERRγ and caused ERRγ-mediated resistance to tamoxifen in ER+ breast cancer cells [96], alluding to the potential of EGFR-ERK1/2 inhibition together with ERR antagonists for hormone-based targeted therapies in patients.

## 3. Conclusions and Perspectives

The evidence summarized here is intended to complement the knowledge on endocrine and metabolic nuclear hormone receptor interactions for cancer immunotherapy (as elaborated in the companion article), with a focus on current preclinical developments. Consistent with the clinical success of established NR combination therapies in patients targeting steroid receptors, future translation of NR ligands addressing exploratory metabolic receptors is a promising avenue. However, adequate translational models must guarantee recapitulation of the geno-to-phenotypes of the human disease. Thus, patient-near-model systems are necessary, including patient-derived tumor organoids (PDOs) or xenografts (PDX). These patient ”avatars” are expected to reduce attrition rates in preclinical drug pipelines and accelerate entry into phase I/II clinical trials.

## Figures and Tables

**Table 1:** Overview of exploratory nuclear receptors in host immunity.

MOA	NR	Ligand *	Type	Function(s) from Preclinical Studies §
Immune activator	RORA/G	Cholesterol	+	RORγt variant promotes adaptive Th17 immunity and inflammation
	NR4A	Cytosporone B	+	Immediate early (“onco”) genes, promote adaptive immunity
	LRH1	Phospholipids	+	Metabolic modifier, promotes adaptive immunity
	HNF4A	Fatty acids	+	Metabolic modifier, promotes adaptive immunity
	CAR	Phenobarbital	+	Xenobiotic sensor and detoxifier, pro-inflammatory
Immune suppressor	PPARB/D	Seladelpar	+	Metabolic modifier, discontinued in human clinical trials
	LXR	Oxysteroles	+	Metabolic modifier, anti-inflammatory
	REVERB	Stenabolic	+	Circadian rhythm transcriptional repressor, anti-inflammatory
	ERRA	Cholesterol	+	Immunomodulator, promotes drug resistance
	PXR	Rifampicin	+	Xenobiotic sensor and detoxifier, anti-inflammatory

* Selected bona fide ligand (agonist = “+”). § Selected functions (https://pubmed.ncbi.nlm.nih.gov; accessed on 1 June 2023) from preclinical studies testing combinations of NR ligands with immune checkpoint Abs (PD1, PDL1, CTLA4, e.a.) and/or interferons in experimental animal models (mice, rats, e.a.) with cancer (solid tumors, leukemia, lymphoma, e.a.) including preclinical studies with NR ligand monotherapy or non-checkpoint combination regimens (chemotherapy, RTK blocking Abs, epigenetic/signaling inhibitors, e.a.).

## Abbreviations

Abs: antibodies, AKT: protein kinase B, AMPK: AMP-activated protein kinase, CAR: constitutive androstane receptor, CAR-T: chimeric antigen receptor T cells, CDK: cyclin-dependent kinase, CK: casein kinase, CTLA4: cytotoxic T-lymphocyte-associated protein 4, DBD: DNA-binding domain, DC: dendritic cells, EAE: experimental autoimmune encephalomyelitis, EGFR: epidermal growth factor receptor, ERK1/2: MAPK3/1, ERR: estrogen-related receptor, GPCR: G-protein coupled receptor, HNF4: human hepatocyte nuclear factor 4, IRAE: immune-related adverse events, JNK/SAPK: MAPK8, LBD: ligand-binding domain, LRH: liver receptor homolog-1, LXR: liver X receptor, MAPK: mitogen-activated protein kinase, MDSC: myeloid-derived suppressor cell, MEK: MAPK kinase, mTORC: mammalian target of rapamycin complex, NK: natural killer cells, NOR/NUR/NURR: nuclear receptor subfamily 4 group A member, NRSF: nuclear receptor superfamily, p38: MAPK14, PD1: programmed cell death-1, PDL1: programmed cell death-1 ligand-1, PI3K: phosphoinositide-3-kinase, PKA/C: protein kinase A/C, PPAR: peroxisome proliferator-activated receptor, PXR: pregnane X receptor, RAF: rat rapidly accelerated fibrosarcoma viral oncogene homolog, RAR: retinoic acid receptor, RAS: Kirsten rat sarcoma viral oncogene homolog, RE: responsive element, REVERB: nuclear receptor subfamily 1 group D member, ROR: retinoic acid receptor-related orphan receptor, RTK: receptor tyrosine kinase, RXR: retinoid X receptor, SRC: steroid/nuclear receptor coactivator, T/B: lymphocytes, TCR: T cell receptor, Tc: cytotoxic T cell, Teff: effector T cell, Th: helper T cell, Treg: regulatory T cell, wt: wildtype.
